# Young onset dementia: implications for employment and finances

**DOI:** 10.1177/14713012221132374

**Published:** 2022-10-18

**Authors:** Caroline Kilty, Suzanne Cahill, Tony Foley, Siobhán Fox

**Affiliations:** Catherine McAuley School of Nursing and Midwifery, 8795 University College Cork, Republic of Ireland; School of Social Work and Social Policy, Trinity College Dublin Centre for Economic and Social Research on Dementia, NUI Galway and Institute of Gerontology, Jonkoping University, Sweden; Department of General Practice, 8795 University College Cork, Republic of Ireland; Centre for Gerontology and Rehabilitation, 8795 University College Cork, Republic of Ireland

**Keywords:** dementia, young onset dementia, caregivers, thematic analysis, qualitative study

## Abstract

**Background:**

People with young onset dementia face unique challenges. Notably, at time of symptom presentation, many people affected by young onset dementia are still employed with significant financial obligations. The aim of this study was to explore the specific impact that young onset dementia has on continued employment and finances and to identify ways to optimise post-diagnostic approaches in this regard.

**Methods:**

Purposive sampling, with a maximum variation technique, was used to recruit a small but diverse range of people with young onset dementia in Ireland. In-depth semi-structured interviews were conducted, and data were analysed using Reflexive Thematic Analysis. In total, 22 interviews were conducted with 10 people with young onset dementia and 12 spouses and children. Two themes were constructed: impact of young onset dementia on (I) employment and (II) finances.

**Findings:**

Participants’ lived accounts showed the devastating effect of a diagnosis of young onset dementia on working life, and the resultant financial, social, and psychological consequences. Participants reported having to leave paid employment early, reported losing contracts and retiring on medical grounds. There were financial implications caused by loss of income, and many additional expenses owing to dementia were incurred. In some families, spouses had to take up employment at the same time as a caring role to mitigate the loss of income, and young children were anxious at the resultant occupational and financial tensions.

**Conclusion:**

People diagnosed with young onset dementia encounter significant challenges associated with employment, and individual and family finances. There is a need for more specific information and guidance from healthcare professionals around employment rights, income support and welfare benefits and the pension status for this group of people. Additionally, healthcare professionals should be cognisant of the additional financial burden people face in young onset dementia when advising on services which incur out-of-pocket costs.

## Introduction

More than 55 million people worldwide are living with dementia (World Health Organisation, 2023). In Western Europe, this number is projected to increase from 9.8 million in 2019 to 18.8 million by 2050 (Alzheimer Europe, 2019). People with young onset dementia (dementia that occurs in people under age 65) represent a small sub-set of all those with dementia, but their challenges are significant, including cognitive, neurological and psychosocial changes (Rabanal, Chatwin, Walker, O’Sullivan, & Williamson, 2018). Reliable epidemiological data on young onset dementia are lacking in Ireland though it is estimated that between 2906 and 4311 people are living with young onset dementia; approximately 1 in 13 of all people with a dementia (Pierse, O’Shea, & Carney, 2019).

In Ireland, service supports for people with dementia have traditionally been designed for older people and based on level of need (Pierse et al., 2019). However, the needs of people with young onset dementia are different, and warrant more specialist approaches (Cahill, O’Shea, & Pierce, 2012). Internationally, there remains a lack of guidance for healthcare professionals who diagnose dementia in younger people (Rabanal et al., 2018) and there is a lack of policy focus for people living with young onset dementia, which has led to significant social and psychological stress for those impacted (Carter, Oyebode, & Koopmans, 2018). The lack of specific services for people with young onset dementia is worrying when we consider the unique impact of dementia on a younger population. Time to diagnosis is longer for people with young onset dementia compared to later onset dementia (dementia diagnosed over age 65), with persons with a younger age of onset waiting up to more than 3 years for confirmation of dementia (Draper et al., 2016; Van Vliet et al., 2013). People living with young onset dementia are often misdiagnosed (Reves, Timmons, Fox, Murphy, & O’Shea, 2018) and symptoms often misattributed as depression or other mental health issues (Department of Health, 2014). Delays in diagnosis can impede access to the necessary information, supports and entitlements which are often only available once diagnosis is confirmed (Hoppe, 2019).

Furthermore, the psychological and social impact is different to late onset dementia as young onset dementia generally occurs at a time in life when people have significant financial, work-related and family-related obligations (Hoppe, 2019; Sikes & Hall, 2018). Having young onset dementia can impact on multiple areas of life not only for the individual but also for families for example, family members’ physical, psychological and emotional health (Svanberg, Spector, & Stott, 2011). Caregivers face changes to relationships and family structures: partners and spouses take on additional parental roles, and children may assume additional caregiving roles (Gelman & Rhames, 2020).

Notably, there may be changes to occupation and financial circumstances (Kimura, Maffioletti, Santos, Baptista, & Dourado, 2015). People with young onset dementia are more likely to be employed in the labour market (Roach, Drummond, & Keady, 2016) and may be required to leave work early because of their symptoms (Silvaggi et al., 2020). Their finances are often adversely affected, and we know that some people experience problems sustaining financial commitments (e.g. mortgage and household payments) (Carter et al., 2018). Caregivers may also reduce work hours or retire early to provide care (Bayly et al., 2021), adding to the social and economic consequences (Carter et al., 2018).

Elsewhere, countries have recently progressed valued strategic change; for example, in the UK, the ANGELA project aimed to improve diagnosis and post-diagnostic support for people with dementia. The authors identified that specialist young onset dementia services performed better, from the provision of support immediately after diagnosis, to continuity of services, care plans and key workers (Stamou, La Fontaine, & Gage, 2021). Information relating to employment, legal and financial matters is critically under-recognised (Mayrhofer, Mathie, McKeown, Bunn, & Goodman, 2018) and much of the information available fails to address the unique needs of people living with young onset dementia (Jones et al., 2018). Notably, there is a lack of research on workplace experiences of dementia (Evans, 2019) which means that people often face complex and precarious personal and financial circumstances (Egdell et al., 2019). Yet more uncommon is the voice of the person affected by these types of challenges.

Although workstreams and service programmes from Ireland’s first National Dementia Strategy (Department of Health, 2014) continue to be rolled out, including new frameworks for diagnostic and post-diagnostic dementia supports, at the time of writing there are no specialist services for Irish people with young onset dementia. Within the Irish literature, there has been little focus on the experience of living with young onset dementia since the works of Haase (2005) and Flynn and Mulcahy (2013). These works showed that people with young onset dementia in Ireland were a neglected and vulnerable group.

The overall research question guiding this study was: what is the lived subjective experience of people living with young onset dementia, and of family members? A specific aim was to develop a deeper understanding of the impact that young onset dementia has on occupation and finances and to identify how to optimise post-diagnostic approaches in this regard.

## Methods

### Design

A qualitative design was employed. In-depth interviews were conducted with people living with young onset dementia, their spouses and children about their experiences. A total of 22 participants were included.

### Setting and participants

A purposive sampling technique was used to recruit a small but diverse range of people with young onset dementia. An information pack was sent via email to national Alzheimer Society (ASI) branches across three regions, including rural and metropolitan areas. Memory clinics were contacted, and the study was also advertised on websites and national social media channels of dementia and healthcare organisations. Potential participants ‘opted-in’ to the study by first telephoning the research team. Eligibility was ascertained during an initial phone conversation and later confirmed face-to-face before data collection. Inclusion criteria for people with young onset dementia were: living with a diagnosis of young onset dementia (self-reported); diagnosed in the previous 5 years. Inclusion criteria for family members were: being a close family caregiver of a person with young onset dementia (i.e. involved in co-decisions about their care); diagnosed in the previous 5 years.

Beyond these broad inclusion criteria, the researcher noted further details during the screening call, including the subtype of dementia, age, relationship of the carer, whether there were young children in the family, and where they were living. This information was used to recruit a diverse purposive sample. Following screening, information leaflets and consent forms were sent to potential participants who opted in and were deemed eligible to participate. Written consent was then obtained prior to interview. In relation to sample size, the authors considered the following points when recruiting and also when determining final sample size. The authors sought to ensure a diverse group was represented (e.g. variation of sub-types dementia) in addition to varied and rich accounts expressed by participants. It is felt that the final sample size is reflective of the focus of the research question and the scope and purpose of the project, considerations in line with reflexive Thematic Analysis (Braun & Clarke, 2021)

### Data collection

Two interview schedules were devised, one for the person with young onset dementia and a second for family members. A priori themes to guide interview topics were derived from the literature on young onset dementia, as well as discussion with an advisory group of clinical and topic area experts, including people living with young onset dementia, family caregivers, advocacy representatives, policymakers, expert academics, and health and social care professionals (HSCPs). Interview schedules were short and consisted of open-ended questions relating to (i) time leading up to diagnosis, (ii) the experience of receiving diagnosis, and (iii) experience of living with young onset dementia.

All but one person who expressed interest in the study were invited to interview. This person had received their diagnosis more than 5 years previously. All interviews were conducted face-to-face in a location convenient to participants (e.g. their home, or a neutral location). Duration was between 30 min and 2 h. All interviews were audiotaped with permission and transcribed verbatim.

People with young onset dementia were given the choice of taking part in the interview individually, or with a spouse, relative or companion. These dyadic interviews were closely monitored by the researchers to ensure no individual experience was overlooked. The inclusive method of dyad interviewing aligns with relationship-centred approach to care, an alternative framework to patient-centred care which recognises the centrality of relationships, families and the community, and how these influence healthcare experiences and outcomes (Soklaridis, Ravitz, Adler Nevo, & Lieff, 2016). It also allows for the opportunity for triangulation of experiences, stories, and accounts of past events (Wilson, Onwuegbuzie, & Manning, 2016).

### Ethical considerations

Two members of the core research team (CK and SF) conducted all interviews; both had prior experience in dementia research. All participants were fully informed about the study’s aims and objectives prior to obtaining written consent, as well as the voluntary nature of the study and scope to withdraw. Relevant guidelines for conducting psychosocial research with people with dementia were followed, including the Alzheimer Europe position paper (Gove et al., 2018) and key publications relating to ethical issues, participatory research, and active involvement of people with dementia in research (Bartlett & Martin, 2002; Dewing, 2007, 2008; Hellström, Nolan, Nordenfelt, & Lundh, 2007; McKeown, Clarke, Ingleton, & Repper, 2010). Ethical approval was obtained from the relevant Research Ethics Committee (Log no 2019-077).

Prior to commencement of the interview, the researchers re-visited the topic of consent, the objectives of the research, and offered participants the opportunity to ask questions. Participants were given contact information for key supports (i.e. Regional Dementia Advisor service, Alzheimer Society National Helpline, Carer’s association numbers).

### Data analysis

Interviews were tape recorded and transcribed. The software nVivo 12 was utilised to support the data management and analysis. Interview data were analysed using reflexive Thematic Analysis Reflexive Thematic Analysis is a flexible, useful research approach which allows for a rich and detailed account of data while also allowing for complexity, and the construction of illustrative and coherent themes (Braun & Clarke, 2021) The process involved searching across interview transcripts to identify repeated patterns of meaning, and to construct illustrative and coherent themes (Braun, Clarke and Hayfield 2022; Braun & Clarke, 2006). The phases of reflexive Thematic Analysis are: (1) process of familiarisation with the data, (2) generating initial codes, (3) searching for themes, (4) reviewing themes, (5) defining and naming themes, and (6) producing the report (Braun & Clarke, 2006, p. 87).

To conduct data analysis, the transcripts were read and re-read, notes were taken in relation to initial observations. When becoming more familiar with the data, labels, or codes, were assigned to areas of importance. Coding was inductive, and was driven by the participants and the accounts they provided (Braun & Clarke, 2021). All transcripts were reviewed in order to identify codes throughout, and were again reviewed and codes collated. Tentative themes were constructed from these codes via a process of contextual decision making, and these themes were again reviewed by cross-checking against the dataset before being determined. A recursive rather than linear approach was followed to allow for scope to move back and forth between steps as the analytic process warranted. Final themes were reviewed and discussed amongst the research team.

### Findings

Fifteen interviews were used to collect data from a total of 22 people (individual = 8; dyadic = 7). Ten participants were diagnosed with young onset dementia. Twelve family members participated, of which eight were spousal carers and four were adult children (18+ years). Participant gender, dementia sub-type and employment status and are shown in Table 1.

**Table 1.** Participant profiles.

Category	*N*		*N*		*N*
People with YOD	10	Spouses	8	Children	4
Female	6	Female	7	Female	3
Male	4	Male	1	Male	1
Dementia sub-type	10				
YOD (specific type unknown or unspecified)	6				
Alzheimer’s disease	2				
Logopenic progressive aphasia^a^	1				
Lewy body dementia	1				
Employment status of PwYOD at time of diagnosis	10				
Stopped work prematurely due to symptoms; without compensation	4				
Formal early retirement due to symptoms/suspected dementia	2				
Unknown	2				
College/volunteer work	1				
Not working	1				

^a^Logopenic progressive aphasia (LPA) is a type of dementia characterized by language disturbance, including difficulty making or understanding speech.

Two key themes were constructed from the findings: (i) impact of young onset dementia on employment, (ii) financial implications of young onset dementia. Participants described the process of obtaining a diagnosis of young onset dementia as challenging. Symptoms often manifested in the workplace and for many, work became unsustainable. Participants described financial implications caused by loss of income, as well as many additional expenses owing to the dementia.

### Theme 1: Impact of young onset dementia on employment

Participants gave accounts of how young onset dementia had impacted upon their employment, particularly in the time leading to diagnosis.

#### Dementia symptoms first apparent at work

Five out of 10 participants reported they were working full-time prior to symptom onset and diagnosis. In several cases, symptoms were first identified at work as they created difficulties in job performance:

*I worked in the health services and I moved ... to the HSE and I discovered that I was making mistakes… I discovered that I hadn’t actually recorded things that I thought I had. I was shocked because I always would’ve been very diligent like that...* [Person living with young onset dementia (Person with young onset dementia) No 4]

Another participant described the stress and embarrassment he experienced when early symptoms became apparent in his work. This man’s wife also talked about the experience:

Person with young onset dementia: *Well… I wasn’t able to do full reports or make a proper decision as such… so I moved from that area then, I just sort of got moved into another area that didn’t involve that type of stress. I didn’t really want to make it... common knowledge either, you know?*

Family member: *None of us recognised it at that stage. I thought he felt* “I have enough, I have 40 years done in a job. I’m 57. I’ve had enough”*…and then he had … very difficult staff to deal with ... They all had issues … one after the other. And in the end he left, I thought too soon. But I think now maybe he just couldn’t cope anymore, really. When we look back, we see things differently now*. [Person with young onset dementia 8 and Family member 10]

Of those working full-time prior to diagnosis, four reported they quit work probably due to health reasons or stress associated with poor work performance. Another was forced to reconsider future plans, including voluntary work and a college course. However, for others having no diagnosis meant that life and work obligations continued despite symptom onset. Several experienced delays in receiving a diagnosis, and these delays meant that critical decisions relating to work, finance and legal issues, were stalled.

*The GP said* “I have no diagnosis (to give you) but I agree it is presenting as a dementia syndrome”*.…But we needed it for work and stuff……If you don’t get your diagnosis, you can’t get your pension…for social welfare, to sort out the invalidity. There wasn’t any emotional connection with (the diagnosis). It was just for legal purposes.* [Family member 5]

#### Precarity of employment status

In discussions about how employment ended, experiences differed. Several participants described the final process of disengagement from work as uncertain. It was often unclear under what circumstances they should leave (e.g. leave on health grounds). Some reported opting for an earlier retirement than they would have liked. In other instances, the employer made the decision about discontinuing work owing to the person’s mistakes and difficulty managing daily workload. One spouse described her husband’s current work status as a mandated period of extended leave, a situation shared by another person with young onset dementia. Some accounts highlighted the absence of employer and peer understanding of dementia, which exacerbated the precarity of their status. In another example, the person had requested to return to work but was awaiting occupational health assessment.

*The occupational (personnel) said that he couldn’t go back but …. We’re still waiting on another assessment about work…She will make an appointment to watch him in work and see is (he) ok…A massive shock, we never expected it at all.* [Family member7]

#### Impact on family members’ employment

Several family members described the impact that dementia had on their own employment circumstances, and the need to balance work roles with caregiving roles, while also taking control of the household situation. For one person this meant retiring from work due to stress:

*I’d still be working…I hadn’t the years to retire. I retired early… and I loved my job…I just decided that the stress was too much, I couldn't cope with the not knowing … not knowing what I’d find when I got home you know. So I just had to give up… I decided that financially and in every other way it would probably be better if I retired. But, that was a big change for me.* [FAMILY MEMBER 10]

For another family member, it meant taking on a new work role when her husband could no longer work due to cognitive impairment.

*He came home one day when his solicitor said something and that’s where I stepped in and got involved in the business. My goal was to keep him working. He had two things in his life, his job and his family. I’ve had a lot of tough years where I’ve given up everything.* [Family member 6]

#### Suggestions for improvement

There was a sense that continued employment could be better supported. Several participants claimed they would have welcomed more information on employment rights and dementia, and reported that rather than finishing work, they would have preferred to continue working with additional supports and adjustments.

*I would like to have still been in the job… maybe (in) a supported way would be a great idea for some people who are a younger age. Because there’s a lot you can do, you know. A lot you still can do, a lot you’ve got to give* [Person with young onset dementia 8]

Another participant, who chose to stop work after receiving her diagnosis, argued that employment advice following diagnosis would have been invaluable.

*If you’re working, can you stay working? …I need someone to come negotiate with me which jobs I can do and which jobs that I can’t do?* [Person with young onset dementia 6]

Diagnosis, or shortly thereafter, was suggested as an opportunity to discuss options around continuing employment.

*I think if we had had (time with diagnosing staff) ….we would have been able to ask questions. We didn’t get to do that…If there was someone there to speak to afterwards, to be able to go to and ask questions. Like our entitlements ... He should still be able to work … He’s a young man… There has to be something.* [Family member 7]

The need for up-to-date practical verbal and written information on employment rights, names of relevant agencies with contact details and next steps forward were highlighted. In this, timing was a key consideration:

*We never got a leaflet, information sheet or any information, nothing leaving that hospital. Even if it’s something that you put in the drawer and look at a month later …. We didn’t get any numbers or anything...you spend so much time afterward ringing around here and there* [Person with young onset dementia 7].

### Theme 2: Financial implications

Participants discussed how the dementia had adversely affected family finances, notably through reduced earnings causing loss or reduction in household income, or additional costs associated with the illness. There were striking examples of challenging symptoms of young onset dementia, including personality changes and impulsive behaviour, which interfered with participants’ ability to manage personal finances.

#### Barriers in accessing support services

In the absence of a confirmed diagnosis, managing family finances and accessing support services was described as challenging. Participants reported considerable difficulty gaining access to financial support, despite employment and income stopping. In most cases, it was the person living with young onset dementia, or a family member, who made initial contact to source specific information and post-diagnostic supports in their area, rather than being supported by Health and Social Care Professionals (HSCPs). The extent to which people living with young onset dementia were left to fend for themselves within the healthcare and social welfare systems was reflected in the following narrative:

*I was referred back to my GP from the memory clinic... And as it turned out I didn’t see anyone again for 4 years. I never got a call back…. there’s no real security there if I had a real problem...* [Person with young onset dementia 6]

Navigating the various systems, offices and requirements was stressful and some participants were denied certain aids because of their age:

*I wanted to see the community health nurse and they said I couldn’t because I wasn’t 65…. I had to fight to see her. The panic button was what I really needed when I was at home alone and I was told that I couldn’t get one because I wasn’t 65. And that’s why I want to push this. It impacts me and it impacts others. That’s the way … that it is. [Person with young onset dementia 6].*

In addition to being refused entitlements on the basis of age restriction, the impact of cognitive impairment was itself a barrier to navigating and instigating relevant entitlements.

*They would say to me like, you need to ring here, you need to do this, you need to do that. And I couldn’t, it went over my head a bit. I kept forgetting parts and pieces… I’m not entitled to a lot of things because I’m not 65. So I’m not entitled to the (homecare) package, I’m not entitled to an alarm, so if I fell I can’t have an alarm because I’m not 65. And there’s a chance that I won’t reach 65.* [Person with young onset dementia 3 (aged 56)]

For one participant, attempting to accessing support was so challenging that she had no option but to circumvent this by contacting local politicians.

*I have a medical card, free travel, but (family) fought for all that…fought for everything. We went to the county counsellor to get help. We know him and he’s helped us for years with little bits over the years. I had to fight for everything. I’m still going to fight for everything. I’m going to get that alarm button in my house as well.* [Person with young onset dementia 3]

#### Direct and indirect costs incurred as a result of the dementia

Apart from reduced income due to changes in employment status, many participants had new expenses associated with dementia which caused financial concerns.

*We have … (to) pay for appointments (and travel) up and down to (hospital). That can be 190 euros for the both of us on the train, that’s the social welfare gone. It’s hard, it is. [Family member 7]*

Some participants recounted how Healthcare professionals had advised numerous measures to ensure safety and well-being for the family, for example, driving assessments and organising counselling for children. This resulted in additional financial strain at a time when financial concerns were already high.

*I was advised that I had to change the gas cooker right away…and I had no money, I went to supplementary welfare, (they said no)... So I didn’t know whether to keep our money for Christmas but I knew it was for safety so I had to change the cooker. And the money…it was down for months and months and months. We were in disaster mode, my friends were going to do a whip around… If we had our savings we would have been grand but that money was gone (impulsively spent by husband with young onset dementia)* [Family member 4].

#### Stress of financial worries on families

A spouse shared the example of her husband, who was later diagnosed with frontotemporal dementia (FTD), arguing with colleagues, beginning to miss time at work and in the end, failing to maintain his contractual commitments. In addition, two other family members described sudden poor decision making leading to financial hardship before diagnosis. In the following case, a father of young children impulsively spent over €20,000, at a time when his employment was already precarious.

*It was unbelievable, the revenue and everything came bang bang bang within a week. … I sat and cried for two weeks, we had no money for (the) future. When they were looking for the money, banging on the doors, he cashed in his life pension….Everything went completely wrong. Not alone was he not in a good place at that time but he stripped us to the bone with our finances...* [Family member 4]

A school-going teenager reported his concerns about family finances. Following his father’s diagnosis, this young man expressed concern about the future. The impact of young onset dementia on family and future security is reflected in the following account.

*What struck me the most was my fear of the future … I was very anxious, and very scared for the financial (situation). I was worried will we be OK? Will we be homeless? Even though my mom told me not to (worry)… I had heard of … how expensive it can be …. He can't work now because of (the dementia). At the time it took us a year to get (financial) help … So we had nothing, we were just every week going down and down and down and down...* [Family member 3]

Barriers with data protection and patient confidentiality added to the complexity of the situation faced by families; in the following example a spouse could not arrange entitlements for her husband even though he had limited awareness of his illness.

*He was on jobseekers (allowance) and I rang them and told them he was in hospital but they said he should be on illness benefit, not jobseekers, but to this day, we never got (entitlements established). There was a new system put in place and I was the 15th person that day (to apply)…(Husband with FTD) said he wasn’t ill. We had a desperate time with GPDR (data prtection/confidentiality…I said you have to talk to me, and they said no…I think the letters should be addressed to both persons in the couple because he was opening the letters and putting them in the bin... he got taken off the list because he didn’t call back within the 5 days…This needs to change.* [Family member 4]

#### Suggestions for improvement

Several participants discussed how helpful an information package might have been in the months following diagnosis. Considering the unique impact that young onset dementia and diagnosis has on the individual and their family, and the work and financial challenges, it was suggested that specialised information and guidance is needed.

*Nobody told me about the invalidity pension. It was a year and one month before we got sorted…The system is wrong, if you had a bit of … guidance. I couldn’t get job seekers, I couldn’t get illness benefit, disability wouldn’t approve us. He should have been on invalidity payment. If someone came in and helped me, if our money was put in place and if we were given proper advice…[Family member 4]*

Participants called for signposting to nominated personnel with a remit in accessing relevant entitlements. Dementia Advisors were cited as key, but in addition, participants stated that they would value the integration of services between healthcare and the offices that process financial supports.

*There should be a letter that goes straight to social welfare that get things sorted straight away. There should be an advocate with the person as well so if they need someone, they (can help) get it sorted. And everything should be instantaneous, there shouldn’t be this fighting for this and that and getting (local politicians) to get people to sort things. You shouldn’t have to fight for this, it’s disgraceful. It’s your diagnosis.* [Person with young onset dementia 3]

## Discussion

Our results extend literature findings on the unique and often very serious impact young onset dementia has on a person’s employment and financial situation. They also contribute to a better understanding of the often-devastating impact young onset dementia has on the family unit. Several participants first noticed problems with their memory or behaviour and mood changes at work and four people quit work prior to their diagnosis. With no definitive diagnosis, no access to Social Welfare entitlements and no work, the majority of those with young onset dementia reported experiencing significant financial strain. Others incurred out-of-pocket expenses because of dementia-related issues. In some few families, spouses had to take up employment at the same time as a caring role, to mitigate the loss of income, and young children were anxious at the resultant occupational and financial tensions. Our findings show that several families were left unsupported following a diagnosis and did not know who to turn to for information and advice about issues such as employment rights, remuneration, and pension status. They would have welcomed clear and timely information about services and entitlements. This was not available for them.

The international literature on young onset dementia and the lived experience for all those affected is gradually burgeoning (Clemerson, Walsh, & Isaac, 2014). There is more awareness of the challenge in obtaining a diagnosis and disclosure (Draper et al., 2016), the important role post-diagnostic interventions play (Aplaon, Belchior, Gélinas, Bier, & Aboujaoudé, 2017) and the overall impact the condition has on family members (Kilty, Boland, Goodwin, & de Róiste, 2019). Our findings suggest that in Ireland, more clarity is needed on employment matters and on the range of financial supports that are available for people with young onset dementia and their families.

The Employment Equality Act (1998–2011) sets out legislation in respect of employment and disability. Employers must take appropriate measures to help a person with a disability perform their work duties, such as adjusting work hours, changing work duties or offering more peer support at work (Government of Ireland, 2011). In keeping with the literature (see Evans, 2019), our findings highlighted how continuing employment remains challenging for both people living with young onset dementia and families. These challenges were particularly profound when the diagnosis was not confirmed, or when the dementia sub-type was atypical (e.g. frontotemporal dementia). In the absence of a confirmed diagnosis communicated to supervisors and co-workers, people living with young onset dementia employed in the labour market may experience difficulties especially in mastering the requirements of their work (Evans, 2019). Previous research has indicated a lack of awareness amongst employers relating to dementia, a lack of training in how to assist employees with dementia, and a lack of commitment to creating relevant policies or sponsoring supportive programs (Egdell et al., 2019; Ritchie, Tolson, & Danson, 2018). Indeed, employment rights are only recognised in a minority of European countries (Alzheimer Europe, 2019).

Participants in this study discussed their diagnosis with their employers to varying extents (if at all, in some cases). According to Williams, Richardson, and Draper (2018), if diagnosis is disclosed to the employer, there is an opportunity to help the person to continue working, and to allow for a longer transition from full employment to discontinuation. It may not always be possible to continue work, but in some cases and depending on the nature of employment, minor adjustments to roles or duties may help (Silvaggi et al., 2020) and employers have been receptive to this (Egdell et al., 2019). Based on findings from the latter study, it seems that employers generally waited until the diagnosis was confirmed, or behaviour had changed before they took action, instead of first having a plan in place (Egdell et al., 2019). Notwithstanding this, people with a diagnosis of young onset dementia are legally entitled to support from employers and if the employee decides to stop work, they should also be provided with the necessary supports to facilitate this (Gibb, O’Caheny, Craig, & Begley, 2019).

Amongst those interviewed in the current study, there was evidence that employment support was lacking. It is apparent that specific information is needed around employment rights, remuneration, and pension status for people who are employed when diagnosed with dementia. As it is, health and social care professionals must navigate fragmented service structures (Vafeas, Jacob, & Jacob, 2020), and the current study findings suggest that in the case of young onset dementia, additional expertise in employment is warranted. Relevant agencies must be prepared for changing demographics and the projected increase in the numbers of people living with young onset dementia (Draper & Withall, 2016). Aside from dementia services, employers, government agencies, health and social care services are well-placed to provide relevant information.

Our findings show that accessing relevant entitlements (e.g. Social Welfare or work-related entitlements) was challenging for many people with young onset dementia and their family members. These findings echo those of Carter et al (2018) who cite financial strains relating to mortgage payments, dependent children and loss of income of others in the home (e.g. spouses). Decrease in income can trigger anxiety within the family and in particular children still at home (Gelman & Rhames, 2020). Roach et al. (2016) found that most of the family caregivers in their study had to retire early from work and were unable to avail of any government supports. Likewise in the current study, several participants expressed facing barriers and waiting long periods for financial assistance, and others not receiving any assistance. As with employment matters, current findings suggest that a delay in diagnosis can impede processes, yet even with a diagnosis, the data here show a lack of clarity within organisations and government agencies (e.g. Department of Employment Affairs and Social Protection). Participants spoke of a lack of support in navigating these processes and being given incorrect advice at times (e.g. told to apply for Illness Benefit when the correct option was Invalidity Pension). In addition,data protection and patient confidentiality added complexity for family members who tried to assist on behalf of the person with young onset dementia.

Staff in existing older adult or dementia services may not have the specialised knowledge to advise regarding financial matters or the relevant agencies responsible. Accounts highlight the need for defined processes for financial supports and entitlements such as discretionary medical cards. Clarity is needed on which entitlements are applicable, how these can be accessed, and what is required to have status approval (e.g. written diagnosis). Employers and staff working in such agencies may benefit from increased awareness around dementia and entitlements. In the current study, participants often took it upon themselves to research the specific information and supports they needed. A key recommendation from these findings is the need for specific guidance for members of the public, healthcare professionals and employers. Such guidance (as has been developed in England and Scotland: Egdell et al., 2019) could improve dementia literacy and enhance clarity on Social Welfare Entitlement, Medical Certification entitlement, and legal matters including the Assisted Decision-Making Capacity Act and Employment rights.

Finally, this study adds to the literature by highlighting the many direct and indirect costs of young onset dementia. There are legal costs associated with updating wills and obtaining Enduring Powers of Attorney, which must be borne sometimes by families already experiencing a reduction in household income. Staff with a remit for diagnosis, as well as ongoing support for people living with young onset dementia, need to be mindful of the financial issues people may be experiencing. Before recommending additional support that will incur costs, staff should advise families about welfare and other financial supports post-diagnosis. Healthcare professionals should then familiarise themselves with supports that are cost neutral; rather than adding additional financial burden, there may be recourse to voluntary agencies or supplementary welfare for household assistance.

More broadly, legal frameworks and participation in decision-making by people with dementia remain overlooked. People living with young onset dementia have historically been excluded and denied the opportunity to participate and engage in service and policy design (Mayrhofer et al., 2018; Rabanal et al., 2018). Dementia is now considered a disability under many countries’ equality acts (Cahill, 2018) and we have seen significant progress in respect of individual autonomy and exercising rights in decision-making (Department of Justice & Equality, 2015). Additionally, human rights law provides for the necessary frameworks for persons with cognitive impairment to request that employers make reasonable adjustments to continue employment (United Nations, 2006). However, Alzheimer’s Europe argue that key questions remain on issues such as legal frameworks to protect the rights of people with dementia in employment, statutory mechanisms for flexible working hours and protected leave for caregivers (Miller & Georges, 2021).

In Ireland, advocacy organisations and dementia experts have been trying to develop models of care that are responsive and that allow for the complexity and individual nature of young onset dementia. Our findings highlight the central importance of information on employment rights and financial support, guidance on appropriate benefits, and the provision of timely, appropriate support from the relevant agencies.

## Conclusion

As the number of people diagnosed with young onset dementia continues to increase, there is an urgent need to plan services for this age cohort. This study highlights some specific areas where service development is needed. Owing to the complexities of young onset dementia and the time of life at which it occurs, people who are employed when diagnosed need specialist advice and information about employment rights, remuneration, and pension status. Findings suggest that delayed diagnosis can contribute to increased uncertainty in work. Timely diagnosis may help with greater work clarity, instigation of supportive processes, work-based reviews and occupational health referrals.

Employers and staff need to be aware of young onset dementia and the correct Human Resources and Occupational Health processes, and policies should developed to support employers. Greater clarity is needed for dementia care workers who assist with diagnosing and signposting. Healthcare professionals need to be aware of the additional financial burden people face because of young onset dementia, and be cognisant of this when providing information and advice. Entitlement processes (e.g. Long term illness cards, medical cards, invalidity pension) should be reviewed and streamlined for those impacted by young onset dementia.
